# Supplementary material for: “Dispense antiretrovirals daily!” restructuring the delivery of HIV services to optimize antiretroviral initiation among men in Malawi
Source: PLoS One. 2021 Feb 22;16(2):e0247409. doi: 10.1371/journal.pone.0247409 (PMC7899340; doi:10.1371/journal.pone.0247409)
Supplement: Attachment — Submitted filename: Letter of Response 2.docx [file pone.0247409.s001.docx]

Dear Editor and Reviewers,

**PONE-D-20-08357: “Dispense Antiretrovirals daily!” Restructuring the delivery of HIV services to optimize antiretroviral initiation among men in Malawi.**

We are thankful for the review over our manuscript which has not only strengthened the message but also enabled us to reflect on the data we had for a better output. The review was thorough and constructive. Please find below our responses to the queries as raised by the reviewers

**Reviewer # 1**

1. The manuscript addresses the important topic of barriers to ART initiation and adherence among men in Malawi. The manuscript is technically sound, but errors in the English and choppy sentence structure make comprehension difficult at times and interrupt the flow of the manuscript. The manuscript would benefit from heavy editing from a professional editor both in terms of English grammar and organization (flow from one sentence/topic to another; particularly in the introduction and discussion sections).

Response: The manuscript has been edited for language and we have revised our paragraphs to highlight topic sentences thereby limiting one idea or only related ideas under one paragraph to promote comprehension. This is reflected mainly in the introduction and discussion sections of the manuscript.

2. The methods section is well-developed according to COREQ guidelines.

Response: We appreciate this comment.

3. Information about ethical approval should also be included in the body of the manuscript, in the methods section.

Response: We have included the ethics statement in the main body as reflected in lines 289-309:

**Ethical Considerations**

Our study obtained ethical approval from Malawi’s College of Medicine Research and Ethics Committee (COMREC- Number P.11/16/2064) prior to study implementation. The District of Health and Social Services (DHSS) for Blantyre and the Director of (Queen Elizabeth Central Hospital (QECH) provided Institutional support for the study to take place in the respective sites. Each study participant provided a written informed consent prior to any study procedures. Participants that could not read nor write thumb-printed on the consent form after it was read to them in the presence of an impartial witness. We assured all participants that their participation in the study would not affect their receipt of medical services at the respective facilities. As a measure of maintaining participants’ confidentiality, anonymity and privacy, we conducted all interviews in a private and quiet room at the facility as per preference of the interviewees. The HIV and ART Coordinators’ interviews were conducted at their office stations as preferred by them. The FGDs were all conducted within a health facility however HIV statuses were not disclosed during the discussion to maintain privacy. The men with an unknown HIV status were referred for HIV testing while those who were HIV infected and not on medications yet were referred for ART as per national guidelines. Interviews with HCWs were conducted after working hours, during lunch breaks and weekends as per participant’s preference to avoid disruption of work. We used codes instead of participants’ names in the study summaries. All audios and transcripts were saved in a password protected computer with limited access to the researchers

4. Line 240: Why were all data collectors female? How might that have affected data collection?

Response: We realise that having only female data collectors may bias our findings, we have acknowledged it as a limitation for the study. This is stated as follows on page:

Lines 802-806: The use of only female data collectors may have limited men from opening up in sharing their views, however, the data collectors were trained on measures of ensuring that men were free to share their insights and also on probing for more information to achieve a comprehensive narrative.

5. In the data analysis section, it would be interesting to provide examples of the thematic codes used.

Response: We have included some examples that led us to realise the thematic codes we presented. See Lines 274-286:

Our three main themes were realised through the collation of similar, related, and recurrent codes, specifically, we grouped all codes on drugs such as dispensation, composition, packaging, and innovations under one overarching theme of differentiating drug dispensing modalities; all codes related to clinic flow, layout and hours and days of operation of a facility were aggregated under Differentiated clinic operations and all codes that referred to the implementation of test and treat and counseling were grouped under the strengthening implementation of available policies theme. We examined each code for further subcategories (40) to ensure that each related concept is grouped under one category and we dropped themes that were not broad enough, for instance, we had a theme on male-specific clinics which we later grouped under clinic operations because it was closely related to that. Fourthly, the research team reviewed the themes and we refined them as appropriate to achieve a correct representation of our findings [

6. Lines 489-495: Includes a quote that was included elsewhere.
   Response: We have deleted the repeated quote and have revised that section.
7. Lines 508-515: It would be helpful to explain what the role of a guardian is in Malawi.

Response: We have included information about a guardian and their role in the Malawian setting in the background to our study and it reads as follows:

Lines 112-116: At the point of ART initiation, a client is supposed to nominate a person who is commonly referred to as a “guardian” who is usually a layperson and could be a friend or family member (32). A guardian is counseled along with a client on the HIV status of the client and information on ARVS to promote adherence and at times to collect the ARVs on behalf of the client

8. Given that men of unknown status, HIV-positive men on ARTs and HIV-positive men not on ARTs were sampled, it would be interesting to note whether there were differences in the insights obtained from each category of men.

Response: We reviewed our data set to ensure that we have correctly represented the views and have highlighted in the narratives areas where various men of different HIV statuses shared their views. These are reflected in lines 362, 377, 427 and 472. Our results should also be considered under the context that men with an unknown HIV status were limited in their comments over other parts of ART services as they have not interfaced with them under their status.

9. Given that the study was conducted in 2017, it would be helpful to explicitly discuss how relevant the findings are in 2020. Has Malawi already rolled-out policy changes that address any of the study's recommendations? If so, is there any evidence that those policy changes have improved uptake of timely and continuous ART by men in Malawi?

Response: We have included this information on the changes that have occurred since then and this is reflected as follows:

Lines 116-124: Despite the lag in men accessing HIV services, there have been no specific HIV service programmes rolled out for men in Malawi. However, anecdotal reports show that some health implementing partners piloted male-friendly clinic services in 12 primary health facilities in Blantyre, Malawi in 2018, a year after we had finalized data collection. The male-friendly clinics would open once a week on Saturday’s targeting newly diagnosed men and those that had problems collecting ARVs during the week. Male- friendly clinics were run by both female and male health care workers providing non-communicable health services, sexually transmitted infection services, HIV testing services, and ARVs dispensation.

Lines 716-718: A similar measure of extending hours of operation in HIV services in Malawi was only applied to HIV Testing services and going forward, it would be important to evaluate this strategy with ART clinics.

Lines 700- 703: Nonetheless, programmatic reports suggest that men advocated for male-only clinics and extensions of hours for them to initiate ARTs which has been tested on a small scale in Malawi and may benefit from further research.

Lines 721-724: Programmatic data on the implementation of male-only clinics in Malawi show that they are effective with the potential for scaling (63).

Lines 740-745: Specifically for Malawi, Multi-Month Scripting (MMS) for stable clients was implemented and clients are given ARVs to last them for six months The goal of MMS is to reduce the number of visits to the clinics for refills which also creates a platform for health facilities to focus more on complex clients that need more attention compared to stable ones. The effectiveness of these changes is yet to be assessed on a larger scale in Malawi (51,66).

Lines 788-791: Malawi is yet to fully implement a verbatim of test and treat approach and this is evidenced by the number of people that are not linked to care instantly which necessitates a review of health system operations to eliminate factors that hinder implementation (70–72).

10. Regarding data availability, there are many participant quotes in the manuscript, but the authors could also make the de-identified transcripts or excerpts of transcripts reflecting the thematic codes underlying the manuscript available on a public data repository.

Response: Currently, the data are still being used in manuscript writing by the authors and students within College of Medicine, After all those commitments, the data will be made available in an open domain. However, the data sets can be made available at reasonable requests from the corresponding author in compliance with College of Medicine’s requirements.

**Reviewer # 2**

1. The paper ‘Dispense Antiretrovirals daily!’ Restructuring delivery of HIV services to optimize ART initiation among men in Malawi is a qualitative study that evaluates the existing health services for men in Blantyre, Malawi. This paper fits into the small but important body of literature from SSA that focuses on HIV service provision for men, a relatively under-researched topic within the HIV literature.
   This qualitative paper is containing findings from an extensive study for which the research team interviewed a range of different health workers and male patients recruited from 7 different clinics in the city. I applaud the authors for being able to get so many rich findings, but unfortunately, I have to reject the paper. Below, I will outline the reasons why I made this decision and will give some ideas on how to move forward.

Response: We appreciate your taking the time to review the paper and have found your comments and suggestions valuable.

2. Firstly, the structure of the background and the rest of the paper is not consistent. In the background, a brief overview of the problem at hand is given, but there is a little description of how the services are structured within the area, what exact services are offered per clinic and how these clinics work organisationally. This is key for the rest of the paper.

Response: We have included a section that discusses on how services are provided and organized in Malawi and agree with the reviewer that this component is key to the paper. The following has been included, see Lines 104 to 124

HIV and ART services in Malawi are provided for free in all Government-owned health facilities and at a cost in private and faith-based organisations. The services include HIV testing, ART initiation, and follow-up in care. Health facilities in the urban areas offer HIV services daily while those in semi-urban and rural areas have a designated day within the week when ART clinics run while HIV testing is offered daily. Malawi has integrated the delivery of HIV services with Tuberculosis, Family planning, and Sexual and reproductive health programmes (30). All services are standardized and follow the same monitoring plan regardless of their location and rarely do facilities experience stock-outs of ARVs (31). At the point of ART initiation, a client is supposed to nominate a person who is commonly referred to as a “guardian” who is usually a layperson and could be a friend or family member (32). A guardian is counseled along with a client on the HIV status of the client and information on ARVS to promote adherence and at times to collect the ARVs on behalf of the client. Despite the lag in men accessing HIV services, there have been no specific HIV service programmes rolled out for men in Malawi. However, anecdotal reports show that some health implementing partners piloted male-friendly clinic services in 12 primary health facilities in Blantyre, Malawi in 2018, a year after we had finalized data collection. The male-friendly clinics would open once a week on Saturday’s targeting newly diagnosed men and those that had problems collecting ARVs during the week. Male- friendly clinics were run by both female and male health care workers providing non-communicable health services, sexually transmitted infection services, HIV testing services, and ARVs dispensation.

3. Ideas around stigma and other barriers to care for men are mentioned, but not adequately unpacked, even though many of the findings point towards stigmatization of men when they come to the clinic. This is a missed opportunity.

Response: We have provided more depth in the narratives of our quotes to shed more on stigmatization and the rationale for the suggested strategies. This is reflected in the results and discussion sections on the following lines: 363-368, 375-380, 388-392, 444-447, 472-479, 572-576, 585-592, 668-675, 676-685, 703-708, 746-751, and 756-766.

4. Then, the methods is too long and too descriptive, and could be shorter. However, this can be done through thorough restructuring and proofreading of the paper.

Response: We believe that our methods section is lengthy mainly because we applied the COREQ guidelines to ensure that it retains the quality as needed for a qualitative paper. This paper is also the first one from our project on HIV and men as such we also wanted it to have the methods laid out in detail so that future papers would refer this paper.

5. In the results, I wanted to congratulate the authors with being able to recruit 15 male health workers, and I was looking forward to reading their remarks on male patient’s behaviours, but this was not provided in detail. Some of the findings section (1.1) were really long, and the quotes were not explained/contextualised within a structured narrative. Others were really short and provided little detail. There are several mentions of ‘a guardian’, even though I thought only men of 18 and above were recruited, which was confusing.

Response: We acknowledge that the minimal clarification on a guardian was confusing and we have included a section in the introduction that clarifies who a guardian is to better contextualize our reference on the same. This is reflected as follows:

Lines 112-116: At the point of ART initiation, a client is supposed to nominate a person who is commonly referred to as a “guardian” who is usually a layperson and could be a friend or family member (32). A guardian is counseled along with a client on the HIV status of the client and information on ARVS to promote adherence and at times to collect the ARVs on behalf of the client

6. A summary of the findings would have been useful and when reading the different sections, I would provide more details on how you interpreted the findings. I would also restructure the paragraphs and re-analyse part of the data, as some quotes are repetitive. I would definitely include a section on Stigma, and on Health System barriers.

Response: We have added explanations regarding the suggestions and have alluded to stigma and health system barriers that may have influenced the participants to suggest the stated strategies. We have however not included a section that specifies barriers as that would be outside the objective of this paper.

We have removed repetitive quotes and have grouped similar ideas under one narrative. The aspects of stigma are alluded as in response to Query Number 3, Reviewer 2

7. In the discussion, little reference is made to existing strategies for men and little advise if given for implementing changes for future practice.

Response: We have included the following statements in the introduction and discussion sections to highlight the existing strategies and advice:

Lines 116-124: Despite the lag in men accessing HIV services, there have been no specific HIV service programmes rolled out for men in Malawi. However, anecdotal reports show that some health implementing partners piloted male-friendly clinic services in 12 primary health facilities in Blantyre, Malawi in 2018, a year after we had finalized data collection. The male-friendly clinics would open once a week on Saturday’s targeting newly diagnosed men and those that had problems collecting ARVs during the week. Male- friendly clinics were run by both female and male health care workers providing non-communicable health services, sexually transmitted infection services, HIV testing services, and ARVs dispensation.

Lines 716-718: A similar measure of extending hours of operation in HIV services in Malawi was only applied to HIV Testing services and going forward, it would be important to evaluate this strategy with ART clinics.

Lines 700- 703: Nonetheless, programmatic reports suggest that men advocated for male-only clinics and extensions of hours for them to initiate ARTs which has been tested on a small scale in Malawi and may benefit from further research.

Lines 721-724: Programmatic data on the implementation of male-only clinics in Malawi show that they are effective with the potential for scaling (63).

Lines 740-745: Specifically for Malawi, Multi-Month Scripting (MMS) for stable clients was implemented and clients are given ARVs to last them for six months The goal of MMS is to reduce the number of visits to the clinics for refills which also creates a platform for health facilities to focus more on complex clients that need more attention compared to stable ones. The effectiveness of these changes is yet to be assessed on a larger scale in Malawi (51,66).

Lines 788-791: Malawi is yet to fully implement a verbatim of test and treat approach and this is evidenced by the number of people that are not linked to care instantly which necessitates a review of health system operations to eliminate factors that hinder implementation (70–72).

Lines 716-718: A similar measure of extending hours of operation in HIV services in Malawi was only applied to HIV Testing services and going forward, it would be important to evaluate this strategy with ART clinics.

Lines 700- 703: Nonetheless, programmatic reports suggest that men advocated for male-only clinics and extensions of hours for them to initiate ARTs which has been tested on a small scale in Malawi and may benefit from further research.

Lines 721-724: Programmatic data on the implementation of male-only clinics in Malawi show that they are effective with the potential for scaling (63).

Lines 740-745: Specifically for Malawi, Multi-Month Scripting (MMS) for stable clients was implemented and clients are given ARVs to last them for six months The goal of MMS is to reduce the number of visits to the clinics for refills which also creates a platform for health facilities to focus more on complex clients that need more attention compared to stable ones. The effectiveness of these changes is yet to be assessed on a larger scale in Malawi (51,66).

Lines 788-791: Malawi is yet to fully implement a verbatim of test and treat approach and this is evidenced by the number of people that are not linked to care instantly which necessitates a review of health system operations to eliminate factors that hinder implementation (70–72).

8. In summary, I think this paper could improve drastically if the authors worked with an independent writing advisor to restructure this paper, as I think the authors put in an incredible amount of work in getting the data and on this important topic. Overall editing is also encouraged to improve the paper.

Response: The paper has been revised and edited accordingly

Sincerely,

Alinane Linda Nyondo-Mipando RNM, Ph.D (Corresponding Author)
