# Supplementary material for: “Dispense antiretrovirals daily!” restructuring the delivery of HIV services to optimize antiretroviral initiation among men in Malawi
Source: PLoS One. 2021 Feb 22;16(2):e0247409. doi: 10.1371/journal.pone.0247409 (PMC7899340; doi:10.1371/journal.pone.0247409)
Supplement: Attachment — Submitted filename: Letter of Response.docx [file pone.0247409.s002.docx]

Dear Editor and Reviewers,

**PONE-D-20-0857R1- “Dispense Antiretrovirals daily!” Restructuring the delivery of HIV services to optimize antiretroviral initiation among men in Malawi.**

We sincerely thank you for taking the time to review our revised manuscript. We have addressed the queries raised and find below our responses.

1. Reviewer #1: Thank you for the revision. The manuscript is much improved, but it still requires a careful edit to improve the English and the flow, facilitate comprehension and to make it as succinct as possible/eliminate redundancies. With substantial editing, I believe it would be fit to publish as the methodology is solid and the results interesting.

**Response: We have had the article edited for language**

2. I began making suggested edits, but it became too time consuming to continue in this way:
   Lines 89-91: There are two ideas presented in this sentence, but I'm not sure that one follows from the other: The services are also biased towards females (21) such that projects among men have primarily focused on men having sex with men (MSM) (22,23).

**Response: We have revised the sentence and it is in two parts now as follows: The services are also biased towards females (21) which has resulted in limited services for men. The services that are available for men have primarily focused on men having sex with men (MSM) and not the general population of men(22,23). Refer to page 4.**

3. Line 115: ARVs instead of ARVS

**Response: This has been updated**

4. Line 150: Delete the second "rate": Blantyre district has the highest prevalence rate of HIV among men at 14% as of...

**Response: The second “rate” has been deleted.**

5. Line 154: IDIs instead of IDIS

**Response: This has been corrected**

6. Lines 155-157: The following sentence is vague; consider rephrasing or deleting: We deliberately sampled men of varying HIV statuses to gather perceptions of all characteristics of men to ensure wider coverage of our findings.

**Response: We have deleted the sentence.**

7. Line 176: Consider changing "after sharing the study and its eligibility, the facility In-charge" to "after explaining the study and the eligibility criteria, the Officer In-charge assisted with the identification of..."

**Response: We have revised the sentence as suggested n line 165**

8. Lines 186-191: I would rephrase as: "We included heterosexual men (as ascertained by asking them their
   sexual orientation) who were 18 years of age and above, able and willing to provide consent, and of a range of HIV statuses: uninfected (confirmed through checking the health passport books), unknown (ascertained by asking if they have ever tested for HIV or not), and HIV infected on ART and not on ART (confirmed through checking the health passport book and/or clinic records)."

**Response: We have revised the sentence as suggested. Refer to lines 175-179.**

9. Line 191: There are places where you are using "facility In-charge" where I think you may mean "Officer In-charge"?

**Response: We have made this consistent by using one term throughout.**

10. Line 207: "assistance from HCWs" instead of "assistance for"

**Response: We have changed it as suggested.**

11. Lines 208-211: Split into two sentences: We included men that were 18 years and above, varying HIV status, on ARVs and not on ARVs, able to provide consent, and willing to participate in the study. These men were identified in the departments within the clinic and the communities around each health facility.

**Response: The sentence has been split into 2 as suggested. Refer to lines 191-193.**

12. Lines 211-212: I would rephrase as: "Men were scheduled for a discussion at a time and place convenient for them."

**Response: The sentence has been rephrased. Refer to lines 192-193.**

13. Lines 219-220: I would rephrase as: With the exception of VM, all authors conducted interviews and all data collectors were female."

**Response: This has been updated as suggested. Refer to lines 199-200.**

14. Line 220: I would add: "Before data collection, ALNM, the Principal Investigator (PI) trained..."

**Response: This has been updated as suggested. Refer to line 200.**

15. Line 221-225: I would rephrase as: "LSK and SS are public health specialists with Masters in Public health. TK has a Bachelor of Nursing and Midwifery and ALNM has a PH.D. in Health Systems and Policy."

**Response: This has been updated as suggested. Refer to lines 202-204**

16. Line 230: Replace "shared of her past research" with "shared her past research"

**Response: The word “of” has been deleted.**

17. Line 234: add an "s" to question

**Response: We have added an “s” to question. Refer to line 213.**

18. Lines 235-238: Were these the broad questions for everyone or just for health care workers? Please clarify.

**Response: We have added that the broad questions were for both men and health care workers. To ease the understanding, we have added the interview and discussion guides as supplementary files.**

19. Lines 246-250: This sentence is a bit choppy and unclear.

**Response: We have revised the sentences and have separated all aspects pertaining to quality under one section. Refer to lines 225-239.**

20. Line 251: Replace "in a language" with "in the language"

**Response: We have revised as suggested. Refer to line 227.**

21. Line 260: NVivo instead of NVIvo

**Response: This has been corrected. Refer to line 242.**

22. Lines 263-265: Suggest rephrasing as: Codes were generated, deductively, from the objectives of the study and also, inductively, from the data.

**Response: We have revised as suggested. Refer to lines 245-246.**

23. Lines 265-267: Suggest editing to: "First, the Principal Investigator listened to the audios and read the transcripts multiple times to gain a deeper understanding of the issues raised"

**Response: We have revised as suggested. Refer to lines 246-248.**

24. Table 1: Overall, the table is a bit confusing and needs improvement. I suggest adding a column for percentages as well as for counts. In terms of education level, those who did not have a primary school education had no education? Or more than a primary school education? Perhaps it would be clearer to show each of the different levels of education represented. Uptake of ARVs is only relevant for those who are HIV-positive, not the full sample of 131 participants, so it is difficult to interpret.

**Response: We have revised the Table and have presented percentages as suggested although we are aware that is not consistent with reporting of qualitative research results. Refer to page 12.**

25. Table 2: Make it clear that the table summarizes the key findings.

**Response: This has been updated. Refer to line 304.**

26. The discussion section and conclusion require editing and focus to improve comprehension and retention of the key contributions of the manuscript.

**Response: We have edited the sections as requested.**

­­­­­­­­­­­­­­­­­­­­­­­­­­­­­­­­­­­­­­­­­­­­­­­________________________________________________________________________________

**Reviewer #2: Comments for the authors:**

1. Thank you so much for giving me the opportunity to review this paper. The structure and writing of this paper has been drastically improved compared to the previous draft. However, I still am of the opinion that the methods section and section 1.1 in the findings is too detailed and can be shortened for readability in the paper. I have also given some suggestions for further readings for the discussion section. Lastly, I would urge the authors to get a professional editor to proofread the publication for grammar, spelling and overall readability of the paper. Please see more detailed comments below. These comments can be addressed with the assistance of the editor.

**Response: Thank you for reviewing our manuscript once again. We have shortened section 1.1 and have had the manuscript edited for grammar, spelling and overall readability of the paper.**

2. Introduction:
   For the following statements, there is there no reference, not even a webpage, health report or policy brief. ‘The implementation of these services must be based on evidence and this is important for your paper However, anecdotal reports show that some health implementing partners piloted male-friendly clinic services in 12 primary health facilities in Blantyre, Malawi in 2018, a year after we had finalized data collection.’ The implementation of these services must be based on evidence and this is important for your paper.

**Response: We have provided the reference, reference number 33**

3. Methods:
   I am still of the opinion that this section is too long and detailed. Although your comment suggest that this is the first publication from the project, it is not a description of the project or M&E paper, therefore there is no need to describe the data collection processes in too much detail.

**Response: We have shortened the methods section as suggested.**

A few more points below which can help you to condense the method section:

4. References on in-depth interviews and other data collection methods are missing (line 131). Identification of participants (both patients and health care workers) for IDI’s and FGD’s could be summarized further. You are loosing the reader here.

**Response: We have summarized these further and have provided the omitted references. The references are inserted in line 132.**

5. Data analysis can be further summarized too, the process is interesting, but written to extensively. take out for instance the following sentence: ‘We examined each code for further subcategories (40) to ensure that each related concept is grouped under one category and we dropped themes that were not broad enough, for instance, we had a theme on male-specific clinics which we later grouped under clinic operations because it was closely related to that.’ (line 282 – 284)

**Response: We have shortened the data analysis section however we have retained some aspects that we were asked to include after the initial review.**

6. Findings:
   I still believe that section one of the findings can be shortened, to improve the flow and structure of the article. Paragraph 1.1 contains too many quotes which makes it too repetitive. Pick a few good ones to make your argument and move on to the next paragraph.

**Response: We have retained those that are quite pertinent to the study.**

7. Also, be very clear about the description of participants and be consistent. The first quote starting on line 365, states ‘younger men FGD participant at Centre 2’. This sentence is incorrect, unless you are quoting 2 men. I would change it to; ‘Male participant FGD at Centre 2’. This is done throughout the article.

**Response: We have retained Younger Men because our FGDs were divided into older and younger men.**

8. Also, is it relevant to know if the participant is infected with HIV or not? And are they honest about it? I would take that out of the descriptions of the quotes as done in line 380.

**Response: This kind of identification was brought it after round 1 review. We now need further direction whether this has to be dropped or left as it is.**

9. At line 390 of the findings, again the description of the health care worker is different, here you say ‘KII, HDA at Centre 3’, please be specific or explain the acronyms at the start of the findings. This is about consistency.

**Response: Please note that all abbreviations have been stated in full at the beginning and we have revised presentation of identifiers to remain consistent.**

10. In Paragraph 1.2, you want to make a clearer connection between the different paragraphs and signpost the reader. An example from line 450, you can add; ‘in addition to dispensing ARV’s on a daily basis, participants also suggested longer opening times for clinics to accommodate work schedules of men.’ The same goes for other paragraphs in the findings section.
    Although this part needs editing and further proofreading, the analysis of the findings and unpacking of the quotes has definitely improved throughout the findings.

**Response: We have had to shorten the quotes used and that part has been take out. We have made connection statements where applicable in the results and discussion section of our manuscript.**

11. Discussion:
    Stigmatization and HIV services has been unpacked accurately in the discussion section. There is a missing reference after the sentence in line 722.

**Response: We appreciate this comment.**

12. The paragraph starting on line 736 gives a good indication of why ARV’s need to be dispensed not per month, but per 3 or 6 months. But there is a need to include that this only works with patients who are stable on ARV’s. In South Africa, stable means being on treatment uninterrupted for at least six months. Another angle to explore can be pill-fatigue, a concept that has come up in similar conversations we had in our research project. Providing treatment for 6 months can prevent pill-fatigue among patients which can lead to non-adherence.

**Response: This was already presented on line 520. We have taken note of pill – fatigue and this has been recommended as part of future studies, considering that if we start on it in the discussion, we may digress from the core objective of the study.**

13. The paragraph starting on line 747 speaks about dispensary of ARV’s in other places than a pharmacy. Here, you can link effectively to studies conducted in South Africa with adherence clubs that meet patients outside of clinic spaces. Medication gets dispensed in community centres or churches, which means patients don’t have to wait in queues, have more freedom and keep their confidentiality. I think referring to some of these studies will improve the discussion section of the paper.

**Reference- We have made reference to a review on Adherence clubs. Refer to line 561.**

14. Conclusion:
    I would suggest not to start with a statement about HIV stigma and services for men, as this was not the focus of your paper. The focus of your paper is how health services can be better tailored for the needs of men.

**Response: We have revised the conclusion and it reads as follows (Lines 623-631):**

**As HIV and AIDS become a chronic disease, health systems need to be restructured to contain the condition into the routine systems with several pathways of accessing treatment that remain convenient and non-discriminatory to the users. Successful initiation and delivery of ARVs to men will require a revision of the service delivery framework that is currently in use in Malawi. Patient centeredness with a focus on the preference of men in accessing ART services is paramount and requires adapting HIV services to the needs of men in their engagement at every level of service delivery, design, and implementation. Elimination of all kinds of stigma especially those unintentionally imposed by the health system will improve the initiation of ARVs among men.**

We remain grateful for the constructive review of our manuscript.

Sincerely,

Alinane Linda Nyondo-Mipando, RNM, Ph.D. (Corresponding Author)
