# Supplementary material for: “Dispense antiretrovirals daily!” restructuring the delivery of HIV services to optimize antiretroviral initiation among men in Malawi
Source: PLoS One. 2021 Feb 22;16(2):e0247409. doi: 10.1371/journal.pone.0247409 (PMC7899340; doi:10.1371/journal.pone.0247409)
Supplement: Attachment — Submitted filename: Letter of Response..docx [file pone.0247409.s003.docx]

Dear Editor,

**PONE-D-20-08357: “Dispense Antiretrovirals daily!” Restructuring the delivery of HIV services to optimize antiretroviral initiation among men in Malawi.**

We are thankful for yet another review over our manuscript which has not only strengthened the message and has highlighted the message we aim to communicate to our readers.

Please find below the responses to the queries raised:

1. Line 33-35: Please remove capital letters in the phrases ‘in-depth interviews’, ‘focus group discussions’, ‘health care workers.’

**Response: This has been corrected. Refer to lines 33-35.**

2. Line 36: Please remove ‘were’.

**Response: This sentence has been revised and now reads as follows: We digitally recorded all the data, transcribed verbatim, managed using NVivo, and analysed it thematically. This is reflected in lines 35-37**

3. - Please check all places where ‘Initiation of antiretroviral drugs’ or similar is mentioned. I would presume it is ‘initiation of antiretroviral treatment’ rather than initiation of drugs.
4. **Response: Thanks you for this insight, it has been corrected wherever applicable in our manuscript.**
5. Please define ‘ART’, ‘ARVs’ at first use, including in abstract

**Response: This has been done**

6. Line 39: Please check the sentence in abstract “Restructuring the delivery and conduct of ART clinics”. Do you intend to imply that ART clinics are delivered?

**Response: Thanks you for this attention to detail as it has clarified the sentence. It now reads as follows in lines 39-40:**

**Restructuring the delivery of antiretroviral (ARVs) treatment and conduct of ART clinics is key to optimizing early initiation of treatment among heterosexual men in Blantyre**

7. Line 43-44. Consider restructuring/punctuating the sentence to enhance logic and ideas flow. It is not easy to understand specifically how ‘removing of other structural barrier’ links into the whole sentence.

**Response: We have revised the presentation and it reads as follows in lines 40-47:**

**The areas requiring restructuring included: Clinic days by offering ARVs daily; Clinic hours to accommodate schedules of men; Clinic layout and flow that preserves privacy and establishment of male-specific clinics; ARV dispensing procedures where clients receive more pills to last them longer than 3 months. Additionally there is need to improve the packaging of ARVs, invent ARVs with less dosing frequency, and dispense ARVs from the main pharmacy. It was further suggested that the test-and-treat strategy be implemented with fidelity and revising the content in counseling sessions with an emphasis on the benefits of ARVs.**

8. Line 45-46: ‘Implementation of test and treat strategy’. I think this phrase could be qualified – is test and treat not being implemented in these facilities/in Malawi?

**Response: We have clarified the sentence and it now reads as follows:**

**It was further suggested that the test-and-treat strategy be implemented with fidelity and revising the content in counseling sessions with an emphasis on the benefits of ARVs. Refer to lines 45-47**

9. Line 39-40. This seems to belong to the conclusion.

**Response: We are proposing to maintain this sentence as it is more of a topic sentence that sets the platform for presenting the results. I tried to remove it and felt that the results section started without a better introduction.**

10. The abbreviation MSM, unless used twice or more, may not need to be included - consider leaving only as ‘men having sex with men’.

**Response: This has been noted and has been removed**.

11. Table 1 revision -please consider moving the absolute number (N=133) from the middle column to percentages column; and have % (N=133); this might read better, if acceptable to the authors.

**Response: This has been done.**

12. Table 1: Please also insert a line in the left column, dividing the age and marital status cells

**Response: This has been done**

13. Line 771: I suggest writing ‘pharmacy’ in small caps

**Response: This has been done**

14. Table 1: Please align all numbers appropriately – as is done in the rows for education, and uptake of ARVs, for example
15. **Response: This has been done**
16. Table 1. “HIV testing’ is written twice

**Response: The repeated words have been deleted**

17. Discussion – Please review and consolidate any points likely repeated between para 1 and para 2; I see what appears repeated reference to integration. Kindly check again

**Response: This has been reviewed and it was not easy to note the stated repetition, however, we have clarified in an area where we are extending on integration. The revision is as follows in lines 526-527.**

**As alluded to earlier, integration of services would be a step towards the implementation of flexible operating rules**

18. The reference to hegemony masculinities - Please confirm that this is the term intended, and that it is how the cited author names it?

**Response: The statement has been revised to avoid misrepresentation on our part and it reads as follows: These male-friendly spaces will also diffuse the notions of viewing oneself as “the real man” resilient to illnesses which inadvertently impedes ART initiation. Refer to lines 560-561.**

19. Table 2: Last line, please remove ‘on’ after ‘emphasizes’

**Response: This has been done.**

20. Table 2: Please remove capital letters where these are not needed in the table text
    - Please also remove capital letters where they are not needed in headings and sub-titles

**Response: The manuscript has been revised and all unnecessary capitalisations have been revised.**

21. Regarding the following query from the authors, please see Academic Editor’s recommendation.
    - Reviewer 2: Also, is it relevant to know if the participant is infected with HIV or not? And are they honest about it? I would take that out of the descriptions of the quotes as done in line 380. Response: This kind of identification was brought it after round 1 review. We now need further direction whether this has to be dropped or left as it is.
    - The Academic Editor reviewed the authors’ response in the first revision and understood that the authors felt including HIV status provided context to the quotes. It is suggested that the authors may retain HIV status but indicate (maybe in the methods section) how this status was determined and any possible limitations, e.g. whether they are confident the HIV status report is authentic.

**Response: We have noted this advice and kindly note that we provided the means of ascertaining HIV status in the methods section.**

**Sincerely,**

Linda A. Nyondo-Mipando RNM, PhD (Corresponding Author)
